# Renal cell carcinoma histological subtype distribution differs by age, gender, and tumor size in coastal Chinese patients

**DOI:** 10.18632/oncotarget.17894

**Published:** 2017-05-16

**Authors:** Junlong Wu, Peipei Zhang, Guiming Zhang, Hongkai Wang, Weijie Gu, Bo Dai, Hailiang Zhang, Guohai Shi, Yijun Shen, Yiping Zhu, Yao Zhu, Dingwei Ye

**Affiliations:** ^1^ Department of Urology, Fudan University Shanghai Cancer Center, Shanghai, 200032, China; ^2^ Department of Pathology, Fudan University Shanghai Cancer Center, Shanghai, China; ^3^ Department of Oncology, Shanghai Medical College, Fudan University, Shanghai, China; ^4^ Department of Urology, The Affiliated Hospital of Qingdao University, Qingdao, China

**Keywords:** renal cell carcinoma, histological subtype, distribution, Chinese, SEER

## Abstract

The distribution pattern of renal cell carcinoma (RCC) histological subtypes according to age, gender and tumor size has not been well illustrated in RCC patients living in fast-developing regions of China. We recruited 2941 patients with clear cell renal cell carcinoma (ccRCC), papillary renal cell carcinoma (PCC) or chromophobe from two hospitals in coastal China (2004−2012) consecutively and draw 538 American Chinese RCC patients’ data with time matched from the Surveillance, Epidemiology, and End Results database. We found that compared with ccRCC patients, chromophobe patients were more likely to be female (OR: 2.538, 95% CI: 1.923−3.350), younger (OR for 51−60 years old: 0.686; OR for over 60 years old: 0.478; reference: age < 50) and to have a larger maximal diameter (Dmax) (OR for Dmax > 7 cm: 1.883; reference: Dmax ≤ 4 cm). Besides, in comparison with coastal Chinese patients, American Chinese individuals had lower Fuhrman grades (*P* < 0.001) and had an onset age 10 years delay. In conclusion, we were the first to observe marked gender, age and tumor size differences in the proportional subtype distribution of RCCs in coastal Chinese patients, and also the first to compare coastal Chinese with American Chinese data.

## INTRODUCTION

Renal cell carcinoma (RCC) is a deadly malignancy [1]. RCC incidence has been shown to be associated with a country's developing level [2]. Although RCC prevalence is relatively low in China as a whole, the southeastern coastal areas, which have a higher gross domestic product, show much higher RCC incidence rates [3]. In 2015, it is estimated that there were approximately 66,800 newly diagnosed cases of RCC and 23,400 deaths in China. The incidence of RCC is higher in urban areas than rural areas, and occurs more frequently in males than in females [4].

There are several RCC histological subtypes that have distinct genetic and clinical features. The most commonly diagnosed subtypes are clear cell RCC (ccRCC), papillary RCC (PCC), and chromophobe [5]. These subtypes are associated with distinct molecular and genetic characteristics [6]. Recent studies suggest that the distribution of RCC histological subtypes is not equivalent in different racial groups [7, 8]. It has been shown that ccRCC is more common in Caucasian populations and PCC is more common in people of African or Afro-Caribbean descent [8]. However, the distribution of RCC histological subtypes in Chinese patients, particularly those from coastal areas, has not been well investigated previously.

The incidence of different RCC subtypes has been epidemiologically shown to be significantly correlated with a patient's geographical location, genetic background, gender, and age [3]. In this study, we examined the distribution ccRCC, PCC, and chromophobe in relation to patients’ age at diagnosis, gender and tumor size in southeastern coastal Chinese patients from two large cancer centers. We also compared our results with American Chinese patients in the Surveillance, Epidemiology, and End Results (SEER) database. These patients should have a similar genetic background to the mainland Chinese patients, and the comparison should help us to determine the impact that social factors and lifestyle have on RCC histological subtype incidence.

## RESULTS

In total, 2941 patients were enrolled in the study, including 2009 patients from FUSCC and 932 patients from Qingdao Cancer Center. Among all the patients, approximately two-thirds of them (67.7%) were male. The median age at diagnosis was 56 years old and median maximal diameter (D_max_) of renal mass was 4.0 cm. Clear-cell renal cell carcinoma took the majority and accounted for 88.9% of all cases. The demographic characteristics of patients are presented in Table 1.

**Table 1:** Patient characteristics of coastal chinese database

Characteristics	FUSCC database (*n* = 2009)	Qingdao Cancer Center database (*n* = 932)	*P* value	Coastal Chinese database (*n* = 2941)	SEER Chinese database (2004–2012, *n* = 538)	*P* value	Total database (*n* = 3479)
Gender, *n* (%)			0.311^a^			**0.017^a^**	
Male	1372 (68.3)	619 (66.4)		1991 (67.7)	336 (62.5)		2327 (66.9)
Female	637 (31.7)	313 (33.6)		950 (32.3)	202 (37.5)		1152 (33.1)
Age at diagnosis (years), median (range)	56 (15–87)	56 (19–84)	0.253^b^	56 (15–87)	64 (19–92)	**< 0.001^b^**	57 (15–92)
Maximal diameter (cm), median (range)	4.0 (0.1–20.0)	4.5 (0.7–20.0)	**< 0.001^c^**	4.0 (0.1–20.0)	4.2 (0.1–29.0)	**< 0.001^c^**	4.0 (0.1–29.0)
Malignant pathology, *n* (%)			0.231^a^			0.218^a^	
Clear cell	1794 (89.3)	820 (88.0)		2614 (88.9)	470 (87.4)		3084 (88.6)
Papillary	97 (4.8)	42 (4.5)		139 (4.7)	35 (6.5)		174 (5.0)
Chromophobe	118 (5.9)	70 (7.5)		188 (6.4)	33 (6.1)		221 (6.4)
Fuhrman Grade, *n* (%)			0.600^a,$^			**< 0.001^a,$^**	
I & II	958 (47.7)	462 (49.6)		1420 (48.3)	319 (59.3)		1739 (50.0)
III & IV	832 (41.4)	384 (41.2)		1216 (41.3)	129 (24.0)		1345 (38.7)
Chromophobe	118 (5.9)	70 (7.5)		188 (6.4)	33 (6.1)		221 (6.3)
Unclear	101 (5.0)	16 (1.7)		117 (4.0)	57 (10.6)		174 (5.0)
^*^Hypertension, *n* (%)			0.192 ^a^		Not provided		
No	832 (69.6)	433 (66.6)		1265 (68.5)			
Yes	364 (30.4)	217 (33.4)		581 (31.5)			
^*^Diabetes, *n* (%)			0.126 ^a^		Not provided		
No	1076 (89.5)	440 (87.0)		1516 (88.8)			
Yes	126 (10.5)	66 (13.0)		192 (11.2)			
Tumor Location, n (%)			0.118 ^a^			0.578 ^a^	
Left	964 (48.0)	485 (52.0)		1449 (49.3)	277 (51.5)		1726 (49.6)
Right	1039 (51.7)	445 (47.7)		1484 (50.4)	259 (48.1)		1743 (50.1)
Bilateral	6 (0.3)	2 (0.3)		8 (0.3)	2 (0.4)		10 (0.3)
^*^Somking Status, *n* (%)			0.065 ^a^		Not provided		
No	716 (80.6)	341 (76.3)		1057 (79.2)			
Ever or present	172 (19.4)	106 (23.7)		278 (20.8)			
Surgical Procedure, *n* (%)			**< 0.001 ^a^**			0.211 ^a^	
Nephron-sparing surgery	558 (27.8)	306 (32.8)		864 (29.4)	155 (28.8)		1019 (29.3)
Complete or radical nephrectomy	1244 (61.9)	575 (61.7)		1819 (61.8)	323 (60.0)		2142 (61.6)
Others or unclear	207 (10.3)	51 (5.5)		258 (8.8)	60 (11.2)		318 (9.1)
^*^BMI (kg/m^2^), median (range)	23.92 (15.06–46.30)	23.78 (17.09–31.83)	0.842 ^b^	23.88 (15.06–46.30)	Not provided		

a. Chi-square test

b. Student's *t* test

c. Mann-Whitney *U* test

*Numbers do not sum to total because of missing values: Hypertension, Diabetes, Smoking status and BMI

$. Comparison of Fuhrman grade distribution, excluding chromophobe and unclear cases.

**Figure 1:**
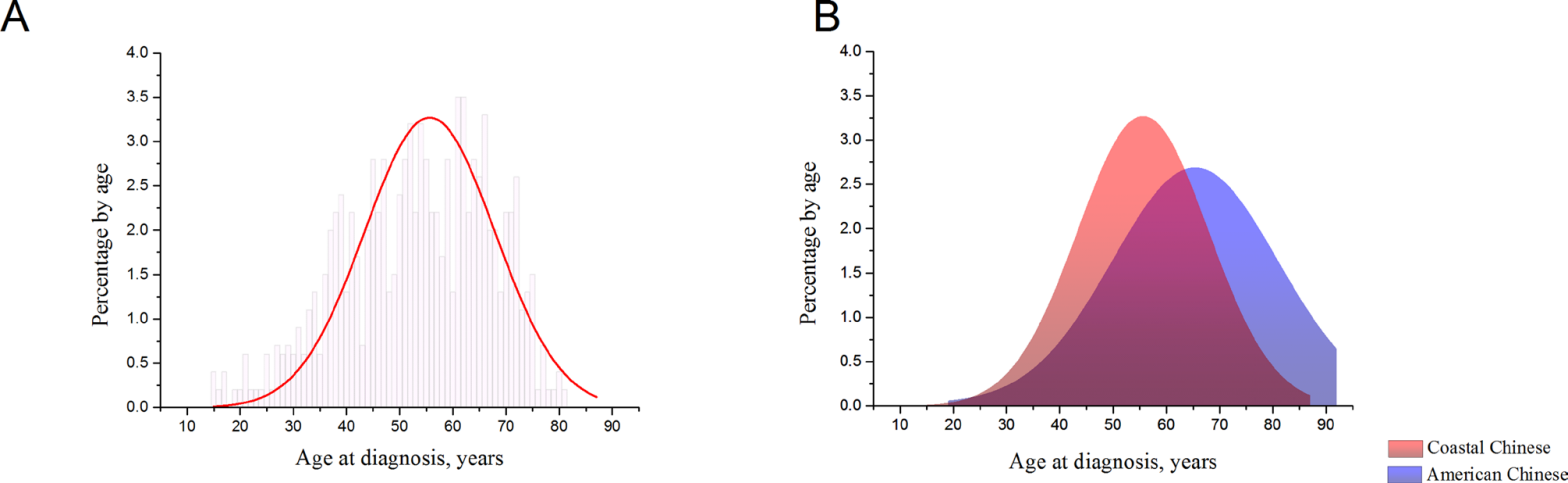
(**A**). Distribution of RCC patients by age at diagnosis in coastal Chinese population. The distribution approaches a normal curve. (**B**). A normalized distribution of the age of onset of RCC in SEER Chinese 2004–2012 database, compared with coastal Chinese patients’ distribution.

Patients from Qingdao Cancer Center had a significantly larger D_max_ (*P* < 0.001). The proportion of different surgical procedure was also different between hospitals (*P* < 0.001). We compared age at diagnosis, gender, histological subtype distribution, Fuhrman grade, hypertension, diabetes, smoking status, tumor location and BMI between patients from two centers as well, but found no statistical significance. Figure 1A shows the distribution of RCC patients by age at diagnosis using a GaussAmp fitting curve (colored red).

We proceeded to analyze RCC histological subtype distribution in Coastal Chinese database according to age, gender, and D_max_ (Table 2). Clear-cell RCC was more common in male patients (90.9% vs 84.7%) while the proportion of patients with chromophobe was higher among females than males (10.3% vs 4.5%). The distribution of RCC subtypes was also significantly different among age and D_max_ groups (*P* = 0.011 and *P* = 0.001, respectively). The proportion of ccRCC patients increased with age increased (*P* = 0.009) and D_max_ decreased (*P* = 0.034). Conversely, the proportion of chromophobe patients decreased with age increased (*P* < 0.001) and D_max_ decreased (*P* < 0.001). No trend regarding the incidence of PCC was observed in different groups.

**Table 2:** Histological type by age at diagnosis, gender and tumor size in Coastal Chinese database

characteristics	No.	Histological type, *N* (%)		
		Clear cell	Papillary	Chromophobe
Gender				
Male	1991	1809 (90.9)	92 (4.6)	90 (4.5)
Female	950	805 (84.7)	47 (4.9)	98 (10.3)
*P* value^a^	**< 0.001**			
Age at diagnosis, years				
≤ 50 yr	1047	912 (87.1)	47 (4.5)	88 (8.4)
51–60 yr	901	801 (88.9)	45 (5.0)	55 (6.1)
> 60 yr	993	901 (90.7)	47 (4.7)	45 (4.5)
*P* value^a^	**0.011**			
*P* value^b^		**0.009**	0.790	**< 0.001**
Tumor size				
D_max_ ≤ 4 cm	1489	1332 (89.5)	74 (5.0)	83 (5.6)
4 cm < D_max_ ≤ 7 cm	985	885 (89.8)	46 (4.7)	54 (5.5)
D_max_ > 7 cm	467	397 (85.0)	19 (4.1)	51 (10.9)
*P* value^a^	**0.001**			
*P* value^b^		**0.034**	0.430	**< 0.001**

a. Chi-square test

b. Cochran-Armitage Trend test.

In addition to coastal Chinese patient cohort, we also investigated RCC in American Chinese patients using time-matched data from SEER database. The demographics of these patients are also shown in Table 1 (right part). Statistical tests were used to compare patients’ available clinical factors between databases. Compared with Coastal Chinese database, SEER database contained more females (37.5% vs 32.3%). American Chinese patients had an approximately 10-year older age of onset (64 vs 56), and they had significantly lower Fuhrman grades (*P* < 0.001). However, the histological subtype distribution was similar between American Chinese patients and coastal Chinese patients.

Table 3 shows the distribution of RCC histological subtypes after multivariate adjustment for age at diagnosis, gender, D_max_, and geographical region. Chromophobe patients were significantly more likely to be female than ccRCC patients (odds ratio (OR) 2.538; 95% confidence interval (CI) 1.923–3.350). Chromophobe patients were also significantly more likely to be younger than ccRCC patients; compared with patients aged ≤ 50 years, the OR for patients aged 51–60 years was 0.686 (95% CI 0.493–0.954) and the OR for patients aged ≥ 60 years was 0.478 (95% CI 0.340–0.671). Compared with ccRCC patients, chromophobe patients were also significantly more likely to have a larger D_max_ (OR for a D_max_ > 7 cm, 1.883; 95% CI 1.340–2.648). No differential PCC or ccRCC subtype association according to age, gender, or D_max_ was found. The incidence of chromophobe and PCC was not associated with different geographical regions.

**Table 3:** Comparison of distribution of sex, age, tumor size and region across histological subtypes of RCC, 2004–2012

	Clear cell N (%)	Papillary *N* (%)	Chromophobe N (%)	Papillary vs clear cell OR (95% CI)	Chromophobe vs clear cell OR (95% CI)
Gender					
Male	2111 (90.7)	115 (4.9)	101 (4.3)	Reference	Reference
Female	973 (84.5)	59 (5.1)	120 (10.4)	1.103 (0.798–1.524)	**2.538 (1.923–3.350)**
Age at diagnosis, years					
≤ 50 yr	1001 (86.8)	53 (4.6)	99 (8.6)	Reference	Reference
51–60 yr	893 (88.7)	49 (4.9)	65 (6.5)	1.034 (0.693–1.542)	**0.686 (0.493–0.954)**
> 60 yr	1190 (90.2)	72 (5.5)	57 (4.3)	1.084 (0.747–1.573)	**0.478 (0.340–0.671)**
Tumor size					
D_max_ ≤ 4cm	1559 (88.9)	96 (5.5)	98 (5.6)	Reference	Reference
4 cm < D_max_ ≤ 7 cm	1028 (89.9)	54 (4.7)	62 (5.4)	0.858 (0.609–1.209)	0.970 (0.697–1.350)
D_max_ > 7 cm	497 (85.4)	24 (4.1)	61 (10.5)	0.767 (0.485–1.215)	**1.883 (1.340–2.648)**
Region					
Coastal Chinese	2614 (88.9)	139 (4.7)	188 (6.4)	Reference	Reference
American Chinese	470 (87.4)	35 (6.5)	33 (6.1)	1.400 (0.954–2.055)	1.026 (0.689–1.526)

**Figure 2:**
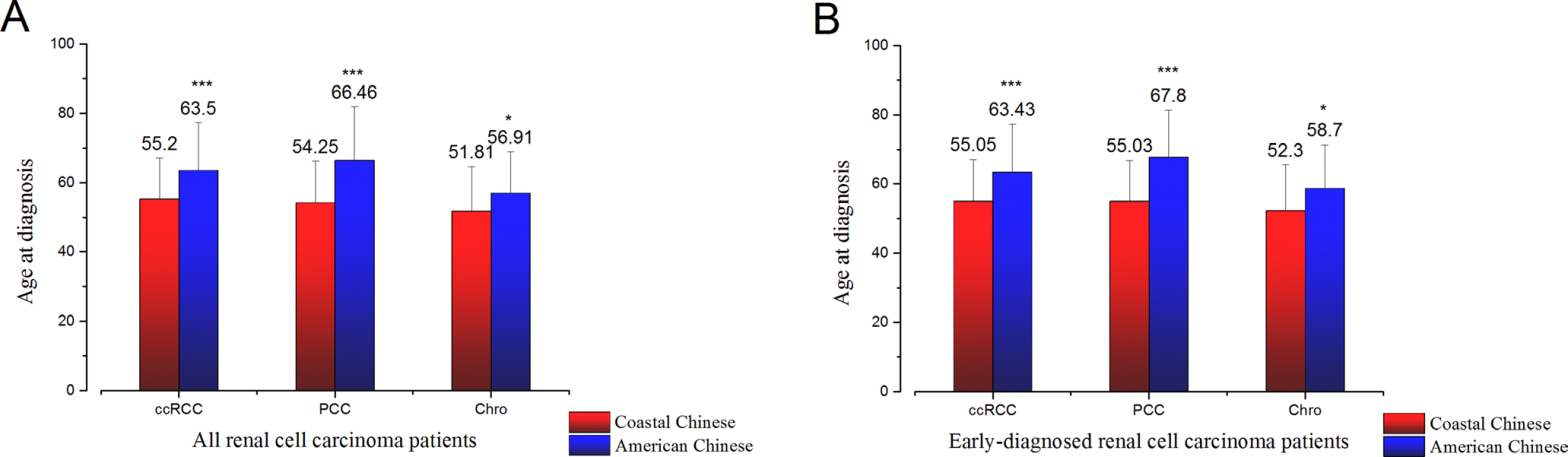
(**A**). Age at diagnosis of three major RCC subtypes of all coastal Chinese patients and all American Chinese patients. (**B**). Age at diagnosis of three major RCC subtypes of early-diagnosed coastal Chinese patients and American Chinese patients (D_max_≤ 7 cm). Mean and standard deviation were plotted. (*p < 0.05; **p < 0.01; ***p < 0.001).

To intuitively compare age at diagnosis, American Chinese data and Coastal Chinese data were GaussAmp fitted together, as shown in Figure 1B. We also compared age of onset between the coastal Chinese and American Chinese patients by histological subtype. For all three RCC histological subtypes, coastal Chinese patients were much younger at diagnosis (Table 4 and Figure 2A). To determine whether early detection resulted in this association, we compared the age of patients with a D_max_ less than 7cm in the coastal and American Chinese patients. Again, we found that early-diagnosed coastal Chinese patients were also younger than American Chinese patients for all three RCC histological subtypes (Table 4 and Figure 2B).

**Table 4:** Comparison of age at diagnosis between Coastal Chinese and American Chinese by histological subtypes

Tumor size	Coastal Chinese Mean (SD)	American Chinese Mean (SD)	*P* value
Overall			
Clear cell	55.20 (11.91)	63.50 (13.72)	**< 0.001**
Papillary	54.25 (11.94)	66.46 (15.37)	**< 0.001**
Chromophobe	51.81 (12.76)	56.91 (11.95)	**0.034**
D_max_ ≤ 7 cm			
Clear cell	55.05 (12.02)	63.43 (13.84)	**< 0.001**
Papillary	55.03 (11.81)	67.80 (13.50)	**< 0.001**
Chromophobe	52.30 (13.23)	58.70 (12.65)	**0.032**

## DISCUSSION

In our coastal Chinese patient cohort, we found that ccRCC was more common in male patients, and the proportion of females with chromophobe was higher than males. The proportion of patients with ccRCC increased as age increased and D_max_ decreased, while the proportion of chromophobe patients had adverse trend. Multivariate analyses indicated Chromophobe patients were significantly more likely to be female, younger, and to have a larger D_max_, compared with ccRCC patients. An approximate 10-year delay in age of onset was observed for American Chinese patients. Furthermore, for all three RCC subtypes, coastal Chinese patients who were diagnosed relatively early also tended to be much younger than their American Chinese counterparts.

Recent studies, while still somewhat limited, corroborate our results regarding the clinical characteristic of RCC in coastal Chinese patients. In 2010, a multi-center study reviewed 1975 RCC cases that occurred in different regions of China [9]. The mean age at diagnosis was 54.5 years, and the study included 1329 male patients (67.3%) and 634 female patients (32.7%). If we only include the three major RCC histological subtypes in our analysis, the majority of patients (90.7%) had ccRCC; PCC and Chromophobe accounted for 5.8% and 3.5% of patients, respectively. Similar studies were conducted in different regions of China recently, achieving similar results [10, 11]. These studies confirm the gender percentage and age at diagnosis for Chinese patients found in our study. However, proportions of PCC and Chromophobe observed in these studies are slightly different to our study. However, studies above included many patients from western and northern China. China is a geographically large country with many different regions of different development levels [12, 13]. In addition to the different levels of development, the genetic background and lifestyle of populations in different regions also varies immensely. To the best of our knowledge, our study is the first to analyze the distribution of RCC histological subtypes in coastal Chinese patients with respect to age, gender, and tumor size, and the first to compare the coastal Chinese data with American Chinese data.

The pathology, genetics, and prognosis of different RCC subtypes are diverse [14]. For example, ccRCC is characterized by silencing of the *VHL* gene and alteration of the hypoxia-inducible factor pathway [15–17]; mutations of the *MET* and *FH* genes are commonly observed in type I and type II PCC, respectively [18, 19]; and abnormal *TP53* and *BHD* genes have been implicated in Chromophobe [20]. Also, previous reports have indicated that different RCC subtypes have different prognoses [6, 21]. So, to gain a better understanding of the genetic and environmental factors that contributed to the subtypes, RCC subtype distribution was investigated in different racial groups [7, 8]. In a study comparing RCC in patients of Caucasian and African or Afro-Caribbean descent, it was shown that Chromophobe patients were significantly more likely to be female than ccRCC patients [7]. As previously reported and results achieved in our study, young women have a higher proportion of Chromophobe compared with ccRCC [22]. It is possible that sex hormones play a role in the development of Chromophobe, leading to the predominance in premenopausal females [23]. Moreover, in other races, it has previously been reported that the odds of having a Chromophobe vs. PCC significantly increase as the tumor size increases, or even versus ccRCC significantly increase when the D_max_ was subdivided according to the criteria in our study [24], which was consistent with our observation. Although many studies have investigated RCC histological subtype risk factors in black or white people, equivalent studies have not been conducted for Asian populations [25]. Our study has provided insights into the distribution of RCC histological subtypes in coastal Chinese patients.

In our study we found that the peak incidence of RCC in American Chinese patients occurred approximately 10 years later than that in coastal Chinese patients. Nowadays, patients with localized RCC typically have no symptoms, whereas are detected during health check-ups [3]. In China, most employees are provided with annual health checks before retirement. However, after retirement, only some individuals continue routine examinations. So the different pattern of annual health checks, could partly explain the early diagnosed age in our coastal Chinese cohort. In addition, environmental or dietary factors may also contribute to this phenomenon. Thus, etiological research is required to more specifically explore the factors that result in this difference.

The limitations of our study include its retrospective nature and the population studied is from select institutions, not population-based. Doing comparison between patients from select hospitals and SEER database leads to bias. In addition, we were not able to obtain complete clinical records regarding the patients’ sporadic RCC risk factors, such as their BMI, hypertension, diabetes, and smoking, TNM stage, Furhman grade, gene-mutation results, and family history [26–28]. We also excluded relatively rare RCC histological subtypes and benign tumors, such as angioleiomyolipoma, Xp11.2 translocation, and some others from our study. Unclassified RCCs were also excluded because they can contain various histological subtypes that could interfere with the data analysis. As such, the distribution of RCC subtypes in Chinese individuals needs to be further validated.

In conclusion, we were the first to observe marked gender, age and tumor size differences in the proportional subtype distribution of RCCs in coastal Chinese patients, and also the first to compare coastal Chinese with American Chinese data. Our results contribute to our understanding of the distribution of RCC subtypes in coastal and American Chinese patients and highlight an interesting difference in the age of onset between populations.

## MATERIALS AND METHODS

### Patients

We enrolled patients diagnosed at the Fudan University Shanghai Cancer Center (FUSCC) and The Affiliated Hospital of Qingdao University from 2004 to 2012. These two cancer centers are located in fast-developing regions of mainland China. The FUSCC and Qingdao Cancer Center serve patients in the south and north of China's southeastern coastal area, respectively. Patients who underwent nephron-sparing surgery, nephrectomy or other surgery to kidney, such as cryosurgery and so on, were recruited consecutively. Patients with benign kidney tumors confirmed by pathology review and patients whose clinical data did not include their age, gender, and tumor size were excluded.

The three most common RCC histological subtypes, ccRCC, PCC, and Chromophobe, included in our study were categorized according to the 2004 WHO classification of renal tumors [29]. Experienced pathologists from the corresponding hospital reviewed each patient's slides. In total, 2941 patients were included in our study including 2009 patients from FUSCC and 932 patients from Qingdao Cancer Center. The present study was carried out in accordance with the ethical standards of Helsinki Declaration II and proved by the Institution Review Board of Fudan University Shanghai Cancer Center. All the patients agreed to participate in the research program and signed informed consent forms.

### SEER Chinese patient data

Urinary cancer data (1973–2012) was downloaded from the SEER website (seer.cancer.org; download date: 2015–12–10). The International Classification of Diseases 10 recode “C64” was used to draw data regarding malignant neoplasms of the kidney, and the SEER race recode “04” was applied to select for Chinese patients. The HISTO3V recodes “8310”, “8260”, and “8317” were then used to filter Chinese patients with ccRCC, PCC, and Chromophobe, respectively. We also restricted the SEER database time-span from 2004 to 2012 to period-match our Coastal database. The SEER database provided us with the histological subtype and complete clinical data of 538 American Chinese patients.

### Statistical analysis

Gender, histological subtype, Fuhrman grade, hypertension, diabetes, smoking status, tumor location and surgical procedure were considered categorical variables, and presented using numbers and proportions. Patients’ age, maximal tumor diameter (D_max_), and body mass index (BMI) were considered continuous variables and reported as median (range). “Percentage by age” was calculated by taking the number of patients diagnosed at a certain age and dividing it by the total number of RCC patients in the database.

“Age at diagnosis” and “percentage by age” were considered as independent and dependent variables, respectively, to perform GaussAmp non-linear fitting (normal distribution fitting) using the formula below (Equation 1):

$$y=y_{0}+Ae^{-\frac{{\left(x-x_{c}\right)}^{2}}{2w^{2}}}$$ (1)

The GaussAmp fitted curves of different databases were plotted using one coordinated system to enable simple visualization and intuitive distribution comparisons. The area under each fitted curve was painted a different color.

Normally distributed continuous data were compared using Student's *t*-test. The nonparametric Mann–Whitney U test was used to compare D_max_ because the data were not normally distributed. The chi-square test was used to compare the distribution of categorical data between groups. Please note that when we compare the distribution of Fuhrman grades between groups, we excluded chromophobe patients because Fuhrman grades were not used for chromophobe patients in these two hospitals based on recommendations [30]. We also used the Cochran–Armitage trend test to examine the distribution of histological subtype as age and D_max_ increased. All tests were two-tailed and P values less than 0.05 were deemed to be statistically significant. SPSS statistic software, version 22.0 (SPSS Inc., Chicago, IL, USA) or SAS version 9.2 (SAS Institute Inc., Cary, NC, USA) were used for data analysis. Figure plotting and GaussAmp fitting were performed using Origin Pro version 9.0 (OriginLab Corporation, Northampton, MA, USA).

## Abbreviations

RCC: renal cell carcinoma
ccRCC: clear-cell renal cell carcinoma
PCC: papillary renal cell carcinoma
SEER: Surveillance, Epidemiology, and End Results
Dmax: maximal diameter
VHL: von Hippel-Lindau
FH: Fumarate Hydratase
BHD: Birt-Hogg-Dubé
